# Anti-snake venom and methanolic extract of *Andrographis paniculata*: a multipronged strategy to neutralize *Naja naja* venom acetylcholinesterase and hyaluronidase

**DOI:** 10.1007/s13205-020-02462-4

**Published:** 2020-10-15

**Authors:** Akshatha Ganesh Nayak, Nitesh Kumar, Smita Shenoy, Maya Roche

**Affiliations:** 1 Department of Biochemistry, Melaka Manipal Medical College (Manipal Campus), Manipal Academy of Higher Education, Manipal-576104, Karnataka India; 2 Department of Pharmacology, Manipal College of Pharmaceutical Sciences, Manipal Academy of Higher Education, Manipal-576104, Karnataka India; 3 Department of Pharmacology, Kasturba Medical College, Manipal, Manipal Academy of Higher Education, Manipal-576104, Karnataka India

**Keywords:** Neurotoxicity, Spreading factor, Multipronged strategy, Supplementary effect, Polyvalent, Local effects

## Abstract

The study investigates the ability of methanolic extract of *Andrographis paniculata* (MAP) to supplement polyvalent anti-snake venom (ASV) in inhibiting neurotoxic enzyme acetylcholinesterase (AChE) and ‘spreading factor’ hyaluronidase from *Naja naja* (N.N) venom. AChE and hyaluronidase activity were measured in 100 or 200 µg of crude venom, respectively, and designated as ‘control’. In Test Group I, enzyme assays were performed immediately after the addition of ASV/MAP/ASV + MAP to the venom. Inhibition of AChE by ASV (100–367 µg) was 12–17%, and of hyaluronidase (22–660 µg) was 33–41%. Under the same conditions, MAP (100–400 µg) inhibited AChE and hyaluronidase to the extent of 17–33% and 17–52%, respectively. When ASV (220 µg) and MAP (100–200 µg) were added together, AChE and hyaluronidase were inhibited to a greater extent from 39–63 to 36–44%, than when either of them was used alone. In Test Group 2, the venom was incubated with ASV/MAP/ASV + MAP for 10–30 min at 37 °C prior to the assay which enhanced AChE inhibition by 6%, 82% and 18% respectively, when compared to Test Group I. Though there was no change in inhibition of hyaluronidase in the presence of ASV, MAP could further increase the extent of inhibition by 27% and ASV + MAP upto 4%. In Test Group III, venom and substrate were incubated for 90 min and hyaluronidase activity was measured after the addition of inhibitors. Here, ASV + MAP caused increased inhibition by 69% compared to ASV alone. The study confirms the ability of phytochemicals in MAP to contribute to a multipronged strategy by supplementing, thereby augmenting the efficacy of ASV.

## Introduction

The World Health Organization (WHO) regards snake bite as a neglected tropical disease (Félix-Silva et al. 2017). According to official figures, India accounts for the highest number of snake bite deaths in the world numbering more than 50 thousand a year ((Mohapatra et al. 2011; Williams et al. 2019). The unofficial number is estimated to be three times more, since most of the deaths in rural areas go unreported (Gupta and Peshin 2012). In India, most venomous bites are attributed to the ‘big four’, i.e., *Bungarus caeruleus* (common krait), *Daboia russelii* (Russell’s viper), *Naja naja* (Indian spectacled cobra) and *Echis carinatus* (saw scaled viper) (Gupta and Peshin 2012).

The venom of *Naja naja* (N.N) is known to contain as many as 81 different toxins (Choudhury et al. 2017) and has systemic effects all over the body (SAM et al. 2009; Kumar et al. 2010; Dissanayake et al. 2018; Berling and Isbister 2015; Ahmed et al. 2008). Neurotoxicity, which is a cardinal feature of envenomation, initially manifests as ptosis of the eyes, progressively spreading to other muscles, finally causing respiratory paralysis and death. Peripheral neurotoxicity of the venom has been attributed to three different toxins. The first, a curare-like peptide also called alpha-neurotoxin, with 60–62 amino acids and molecular weight of 6–7 KDa, binds to the post-synaptic nicotinic acetylcholine receptors in the neuromuscular junction (Barber et al. 2013). This prevents the binding of acetylcholine (ACh) to the neuromuscular junction, blocking neurotransmission. The second, phospholipase A_2_, attacks the lecithin in all membranes including those of the neurons (Paoli et al. 2009; Urs et al. 2014) and subcellular organelles causing irreversible destruction of cells. The third, acetylcholinesterase (AChE), blocks neuromuscular transmission by destroying ACh in the synaptic cleft. (Ranawaka et al. 2013). It is well established that elapid venoms including different species of Naja contain high amounts of AChE, with molecular weight of 67 ± 2 KDa (Raivo Raba et al. 1979) and resemble the human AChE in catalytic activity. It occurs as a nonamphiphilic monomer and hydrolyzes acetylthiocholine faster than propionylthiocholine and butyrylthiocholine (Frobert et al. 1997). Blocking venom AChE would enhance the availability of ACh in the neuromuscular junction and relieve paralysis. Neostigmine used in the treatment of snake bite works on a similar principle (Anil et al. 2010; Naphade and Shetti, 1977; Lee et al. 2004).

The ‘spreading factor’ hyaluronidase (molecular weight—70.4KDa), present in N.N venom (Kemparaju and Girish 2006), allows the spread of venom toxins by digesting hyaluronic acid in the extracellular matrix (ECM) (Urs et al. 2014; Girish et al. 2004). This increases the binding of toxins to target tissues and is one of the factors indirectly causing deleterious local and systemic effects (Kemparaju and Girish 2006). When N.N venom was injected into mice followed by the administration of purified hyaluronidase inhibitors, the survival time was prolonged (Girish and Kemparaju, 2006). So, an antitoxin which can neutralize hyaluronidase would minimize local and systemic toxicity and ensure a better outcome in N.N bite victims.

At present, in India, envenomation by N.N is treated with a polyvalent anti-snake venom (ASV), which contains antibodies against the venom of the ‘big four’ snake species, as mentioned above. ASV in India is only partially purified, expensive and its use is associated with a multitude of allergic reactions. It can also trigger anaphylaxis leading to death (Chube et al. 2016; de Silva et al. 2016). Higher doses of ASV have been correlated to greater risks of anaphylactic reactions (Chube et al. 2016). It is well known that ASV cannot neutralize bound toxins (Sarin et al. 2017; Chube et al. 2016; Bawaskar and Bawaskar 2015). Also, systemically administered ASV is less efficient in penetrating into the bite site (Girish and Kemparaju 2005; Rucavado et al. 2004). Thus, while the ASV does save lives, mitigating the local effects of the venom to ensure the rescue of affected tissues requires an alternative strategy of supplementation of ASV with a suitable antitoxin.

*Andrographis paniculata* (A.P), also called ‘King of bitters’, is a herb which has been used by traditional healers in the treatment of snake bite all over South Asia, including India (Gopi et al. 2011; Samy et al. 2008). In vitro studies with the methanolic extract of A.P (MAP) has been reported to inhibit N.N venom AChE and hyaluronidase (Sivakumar and Manikandan 2015; Gopi et al. 2011). However, in these in vitro studies with MAP, venom and MAP were pre-incubated in an effort to inhibit the enzymes of interest, after which the residual enzyme activity was reported as percent inhibition. These experiments cannot be extrapolated to a real-life scenario, where the snake bite occurs first, following which ASV (containing the putative inhibitors) is administered. When ethanolic extract of A.P was administered intraperitoneally to envenomed mice, a significant increase in mean survival time was observed, but death could not be prevented (Premendran et al. 2011). To justify the use of plant-derived antitoxins and their comparison with ASV, a modified methodology was adopted in a thromboelastographic study of hemostatic abnormalities caused by N.N venom, in which the venom was allowed to act on the blood first, following which the ASV or MAP or their combination was added. These studies measured clotting in real time and proved the ability of the MAP as a supplement to ASV in completely normalizing hemostatic abnormalities caused by N.N venom (Nayak et al. 2020). The present in vitro study conforms to the same modified methodology, focusing on AChE and hyaluronidase, to explore how the MAP stacks up against the standard treatment for N.N bite, i.e., ASV. It also evaluates whether the supplementation with MAP has a better outcome in the presence of lower concentrations of ASV.

## Materials and methods

### Ethical clearance

The present study was conducted after obtaining permission from the Institutional Ethics Committee (IEC-320/2017), Kasturba Medical College, Manipal Academy of Higher Education, Manipal, India.

### *Naja naja* venom

N.N venom as lyophilized powder was procured from K.V Institute, Ballia, Uttar Pradesh, India, and 10 mg of venom was reconstituted in 1 ml of 0.08 M phosphate buffer (pH 7.6). Aliquots of working venom solution were prepared using the same buffer. N.N venom was used at a concentration of 100 µg for the estimation of AChE activity and 200 µg for hyaluronidase activity.

### Anti-snake venom

Lyophilized polyvalent ASV was procured from Bharat Serums and Vaccines Ltd, Ambernath, Maharashtra, India. The entire contents of the vial (22 mg in terms of Lowry’s protein) were reconstituted using 10 ml sterile water provided by the manufacturing company and stored at 2–8 °C. According to the manufacturer, each ml of the ASV could neutralize 0.6 mg of N.N venom. Each ml of reconstituted ASV contained 2.2 mg protein by Lowry’s method (Randall and Lewis 1951). Aliquots of reconstituted ASV used in experiments with AChE were in the range of 50 µl (110 µg) to 167 µl (367.4 µg) and for hyaluronidase in the range of 10 µl (22 µg) to 300 µl (660 µg). The values in parentheses represent protein in the ASV based on Lowry’s method.

### *Andrographis paniculata*

The MAP used in this study was obtained from Natural Remedies Private Limited, Bangalore, and was previously characterized by GC–MS analysis (Nayak et al. 2020). MAP was dissolved in dimethyl sulfoxide (DMSO-99% pure). Stock solutions of MAP containing 1 mg, 2 mg and 4 mg per ml of DMSO were prepared and aliquots containing 100 µg, 200 µg and 400 µg respectively, were used for the estimation of AChE and hyaluronidase.

### Qualitative analysis of MAP for the presence of phytochemicals

Preliminary phytochemical analysis of MAP was carried out for the presence of flavonoids, phenols, carbohydrates, alkaloids, tannins and terpenoids using standard protocols (Sivakumar and Manikandan 2015; Aziz and Iqbal 2013).

### Quantitative analysis

Quantitative analysis of MAP was performed for total phenolic content using Folin–Ciocalteu’s method with gallic acid as standard and represented in terms of milligrams of gallic acid (Rai et al. 2018) equivalents (GAE) per gram (mg GAE/g) of the extract. Total flavonoid content in the extract was determined using quercetin as standard (Saxena and Jain 2018) and represented in terms of milligrams of quercetin equivalents (QE) per gram (mg/g) of extract.

### Estimation of AChE activity

**Fig. 1:**
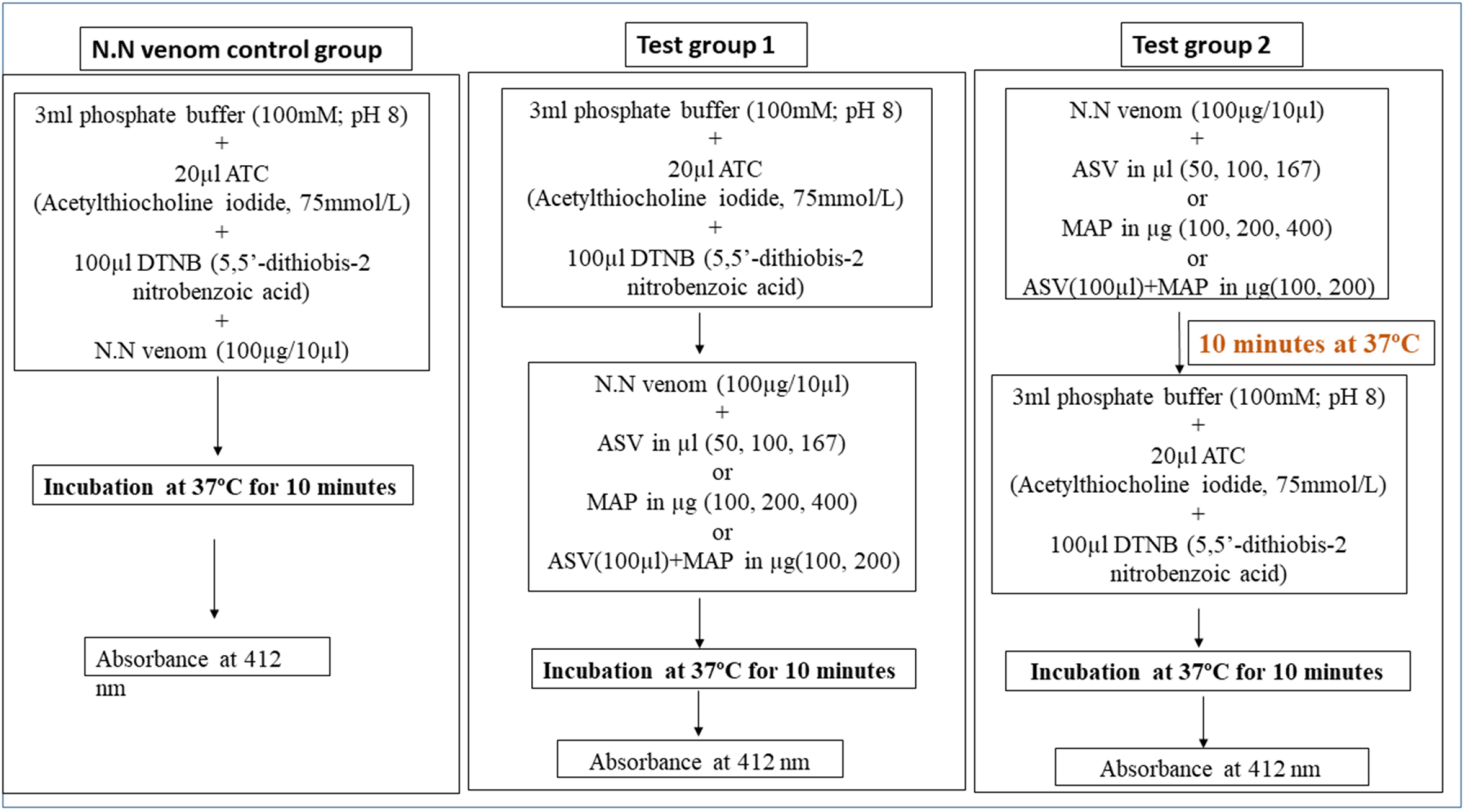
Effect of ASV or MAP or combination of ASV + MAP on acetylcholinesterase (AChE) activity of N.N venom. The flowchart represents the methodology followed during estimation of N.N venom AChE activity in different groups, i.e., N.N venom control group, Test group 1 and Test group 2. ASV: anti-snake venom; MAP: methanolic extract of *Andrographis paniculata*; AChE: acetylcholinesterase; N.N: Naja naja

AChE activity was assayed by Ellman’s method (Ellman et al. 1961). For N.N venom control group, venom was added to the reaction mixture containing substrate, acetylthiocholine iodide (ATC) and 5,5-dithio-bis-(2-nitrobenzoic acid) (DTNB), in the presence of phosphate buffer and incubated for 10 min at 37 °C. Absorbance was measured at 412 nm (Fig. 1). AChE activity of 100 µg N.N venom (control) was considered as 100% and its inhibition by ASV or MAP or combination of the two was studied by adding different concentrations of ASV, MAP or combination of ASV and MAP to the reaction mixture.

In Test group 1 (Fig. 1), N.N venom and different concentrations of ASV/MAP/ASV + MAP were added to a reaction mixture containing ATC, DTNB and phosphate buffer. There was no time gap between the additions of successive reagents. The reaction mixture was incubated for 10 min at 37 °C and absorbance was measured at 412 nm. This set of experiments was carried out in an effort to ascertain the immediate effect of ASV or MAP or the combination of the two in inhibiting AChE activity.

In Test group 2 (Fig. 1), after the addition of ASV or MAP or their combination to the venom, the mixture was incubated for 10 min at 37 °C, to ensure complex formation between AChE of the venom, the ASV and phytochemicals in MAP. This was followed by the addition of ATC, DTNB and buffer and was incubated for another 10 min after which AChE activity was measured.

### Estimation of hyaluronidase activity of N.N venom

**Fig. 2:**
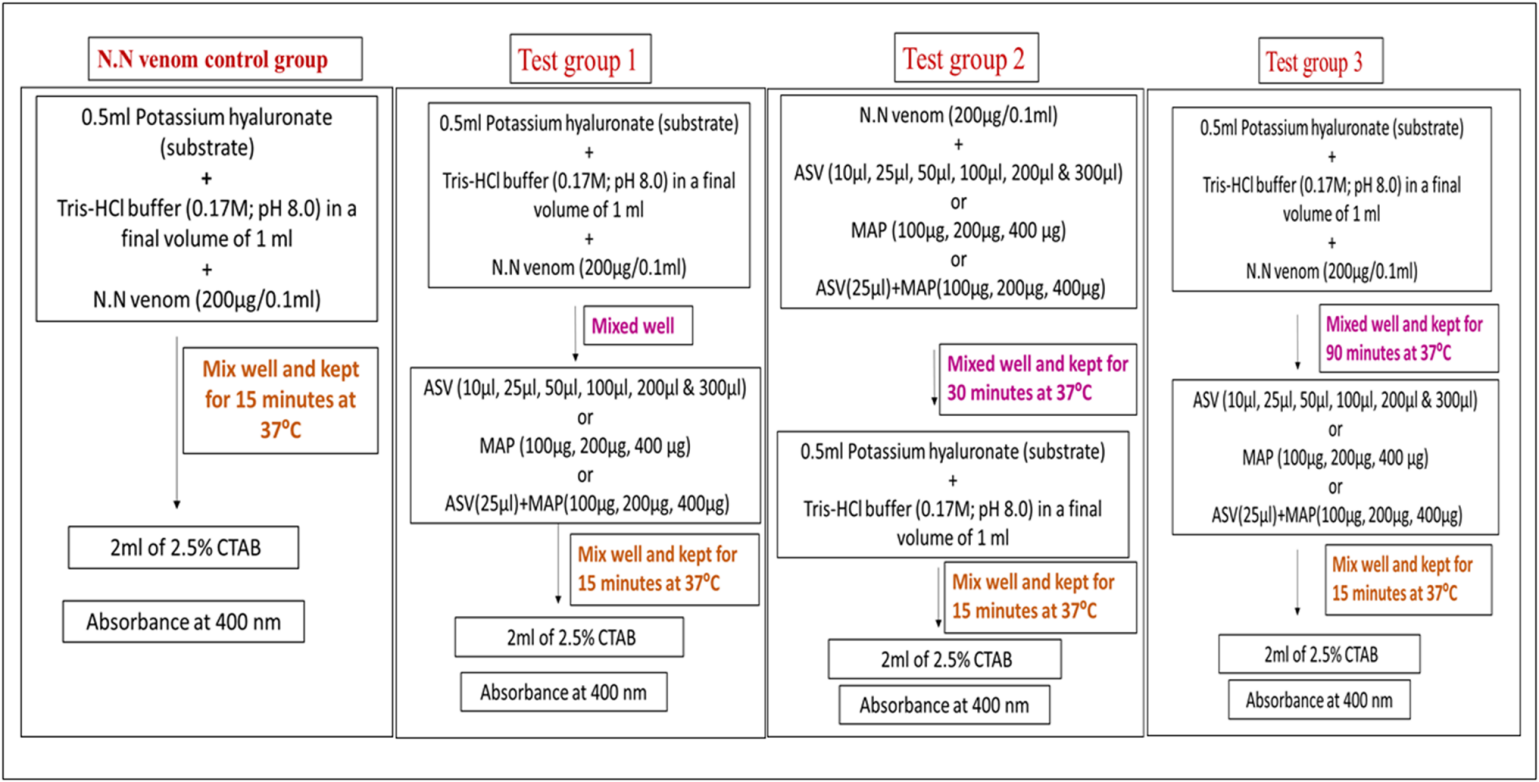
Effect of ASV or MAP or combination of ASV + MAP on hyaluronidase activity of N.N venom. The chart represents the details of the experiment conducted during the estimation of effects of various concentrations of ASV or MAP or ASV + MAP on N.N venom hyaluronidase in different groups, i.e., N.N venom control group, Test group 1, Test group 2 and Test group 3; ASV: anti-snake venom; MAP: methanolic extract of *Andrographis paniculata*; N.N: Naja naja; CTAB: cetyltrimethyl ammonium bromide

The hyaluronidase assay of crude venom was performed turbidimetrically using potassium hyaluronate as a substrate (Pukrittayakamee et al. 1988; Sivakumar and Manikandan 2015). The substrate was dissolved in Tris–HCl buffer 0.17 M, pH 8.0. For N.N venom control group (Fig. 2), the reaction mixture contained 250 µg hyaluronic acid, 200 µg N.N. venom and Tris–HCl buffer, 0.17 M. pH 8.0 in a final volume of 1.0 ml. The mixture was incubated for 15 min at 37 °C and the reaction was stopped by the addition of 2 mL of 2.5% (w/v) cetyltrimethylammonium bromide (CTAB) in 2% (w/v) NaOH. The absorbance was read at 400 nm. The decrease in the turbidity is proportional to hyaluronidase activity and is measured in terms of decrease in the absorbance. Hyaluronidase activity of 200 µg N.N venom was considered as 100% and its inhibition by ASV or MAP or their combination was recorded in three groups of experiments.

In Test group 1 (Fig. 2), N.N venom (200 µg/0.1 ml) was mixed with substrate and Tris–HCl buffer, followed immediately by the addition of different concentrations of ASV/MAP/ASV + MAP in a final volume of 1 ml. This experiment assesses the extent of the reaction between hyaluronidase and its putative inhibitors and also whether it was a swift reaction.

In Test group 2, N.N venom (200 µg/0.1 ml) was incubated with different concentrations of ASV/MAP/ASV + MAP for 30 min at 37 °C, to allow the complex formation between the venom enzyme and components of ASV and MAP. This was followed by the addition of substrate and Tris–HCl buffer to the reaction mixture in a final volume of 1 ml and processed as shown in Fig. 2.

In Test group 3, the digestion of the extracellular matrix by the venom is a time-dependent process which must be assessed over a prolonged period of time. This was evaluated by incubating the venom with the substrate for 90 min in the presence of buffer. This was followed by the addition of ASV/MAP/ASV + MAP and processed as shown in Fig. 2.

### Statistical analysis

All the values are represented in terms of mean ± SEM for triplicate samples. The data were analyzed using one-way ANOVA, followed by Tukey’s post hoc test by SPSS software 16.0. *p* < 0.05 was considered as statistically significant. Inhibitory concentration 50 (IC_50_) of ASV and MAP was calculated by regression analysis using Microsoft Excel.

## Results

### Qualitative analysis of MAP (phytochemical analysis)

Phytochemical analysis of MAP revealed the presence of flavonoids, phenols, carbohydrates, tannins, terpenoids in MAP and absence of alkaloids.

### Quantitative analysis of MAP

Total phenolic content was 43.55 mg GAE/g of extract and total flavonoids were 11 mg QE/g of exctract.

### AChE activity

**Fig. 3:**
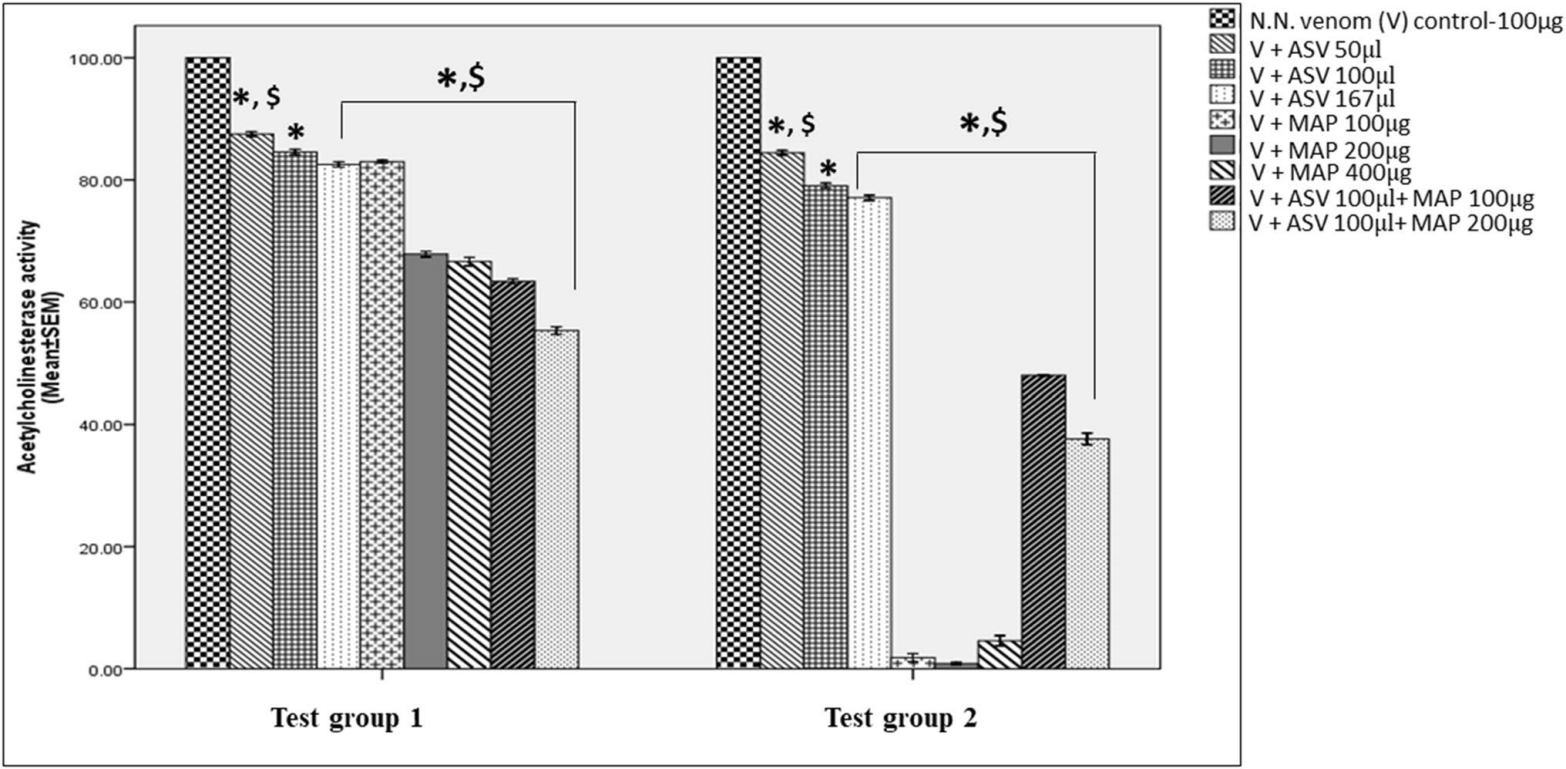
Inhibition of N.N venom acetylcholinesterase (AChE) activity. Comparison of AChE activity between Test group 1 and Test group 2 where Test group 1 represents estimation of AChE in N.N venom after immediate addition of ASV or MAP or combination of ASV + MAP to the substrate. There was no time gap between the additions of successive reagents, and Test group 2 represents estimation of AChE in N.N venom, where ASV or MAP or combination of ASV + MAP was incubated with N.N venom for 10 min at 37 °C prior to the addition of the substrate; all values represent mean ± SEM of three samples, where *p < 0.05 compared to N.N venom control which was considered 100%; ^$^*p* < 0.05 compared to [V (100 µg) + ASV (100 µl)]; *AChE* acetylcholinesterase, *N.N* *Naja naja*, *ASV* anti-snake venom, *MAP* methanolic extract of *Andrographis paniculata*, *V* *Naja naja* venom

According to the manufacturer, 167 μl (367.4 μg Lowry protein) is the concentration of ASV required to neutralize 100 μg of N.N. venom. At this concentration, the inhibition of AChE activity (Test group 1) by ASV (IC_50_-689,959.6 µl) was poor, being only to the extent of 17% (Table 1). Incubation of the venom with ASV prior to the assay for 10 min (for facilitating complex formation, Test group 2, IC_50_-16,425.3 µl) increased the inhibition of the enzyme only marginally, by 5.5%. Inhibition of AChE activity by MAP (Test group 1, IC_50_-1292.8 µg) was concentration dependent. Immediate addition of MAP to the venom was more efficient than immediate addition of ASV in inhibiting AChE activity (Table 1). Maximum inhibition was observed at 200 µg with no further increase in inhibition at 400 µg. Decreasing the concentration of ASV by 40% and supplementing it with MAP showed much better results compared to ASV alone. A comparison of ASV (100 μl) + MAP (200 μg) to ASV (100 μl) alone improved AChE inhibition by 66% (Fig. 3). A comparison of ASV (100 µl) + MAP (200 µg) to MAP (200 µg) alone showed an increase in inhibition by 26%.

**Table 1:** Effect of ASV or MAP or combination of ASV + MAP on acetylcholinesterase (AChE) activity of N.N venom

	Test group 1 Percent inhibition in AChE activity (mean ± SEM)	Test group 2 Percent inhibition in AChE activity (mean ± SEM)	Increase in percent inhibition in AChE activity: comparison of Test group 2 to Test group 1
Venom (100 µg) + ASV 50 µl (110 µg^@^)	12.29 ± 0.19^$^	15.6 ± 0.19	3.3
Venom (100 µg) + ASV 100 µl (220 µg^@^)	15.04 ± 0.22	20.75 ± 0.13	5.71
Venom (100 µg) + ASV 167 µl (367.4 µg^@^)	17.06 ± 0.21^$^	22.58 ± 0.06^$^	5.52
Venom (100 µg) + MAP (100 µg)	16.85 ± 0.12^$^	98.77 ± 0.12^$^	81.92
Venom (100 µg) + MAP (200 µg)	32.60 ± 0.23^$^	99 ± 0.01^$^	66.4
Venom (100 µg) + MAP (400 µg)	33.36 ± 0.48^$^	95.26 ± 0.01^$^	61.9
Venom (100 µg) + ASV100µl (220 µg^@^) + MAP (100 µg)	36.45 ± 0.20^$^	51.89 ± 0.07^$^	15.44
Venom (100 µg) + ASV100µl (220 µg^@^) + MAP (200 µg)	44.17 ± 0.3^$^	61.78 ± 0.03 ^$^	17.61

Test group 1 represents estimation of AChE in N.N venom after addition of ASV or MAP or combination of ASV + MAP to the substrate. There was no time gap between the additions of successive reagents; Test group 2 represents estimation of AChE in N.N venom where ASV or MAP or combination of ASV + MAP were incubated with N.N venom for 10 min at 37 °C prior to the addition of the substrate; AChE activity of 100 µg N.N venom was considered as 100% and designated as venom control. All the test group values of AChE activity were compared with venom control. Test group values shown above represent mean ± SEM of triplicate samples where ^$^*p* < 0.05 compared to Venom (100 µg) + ASV (100 µl); ^@^represents concentration of proteins in ASV based on Lowry’s method

*N.N* *Naja naja*, *MAP* methanolic extract of *Andrographis paniculata*, *ASV* anti-snake venom, *AChE* acetylcholinesterase

In Test group 2, incubation of venom with MAP even at 100 μg concentration for just 10 min almost completely neutralized AChE activity. This is in contrast to ASV, which under the same conditions was only marginally effective to the extent of only 22.6%. A comparison of ASV (100 μl) + MAP (200 µg) to ASV (100 µl) alone showed improved inhibition of AChE by 64% (Fig. 3). There was a decrease of 38% in inhibition when MAP alone (200 µg) was compared to ASV (100 µl) + MAP (200 µg). In general, incubation of N.N venom with ASV/MAP/ASV + MAP was found to be more effective in inhibiting AChE than their immediate addition to the reaction mixture at all concentrations.

### Hyaluronidase activity

ASV required to neutralize 200 µg N.N venom was stated to be 333.3 µl (according to the manufacturer). In Test group 1 (Table 2), ASV (IC_50_-19,004.9 µl) at 50 µl (110 µg) caused maximum inhibition of hyaluronidase activity (*p* < 0.05, 39.4%), with no further increase in inhibition thereafter, on increasing the ASV concentration up to 300 µl. Thus, ASV was only partially effective against venom hyaluronidase. In Test group 2, incubation of ASV (IC_50_-3518.6 µl) with venom for 30 min prior to the addition of substrate did not produce any change in the degree of inhibition compared to Test group 1, indicating that the reaction of hyaluronidase with the ASV is swift and not time dependent. In Test group 3, there was a drastic decrease (upto 56%) in inhibition of hyaluronidase activity at ASV 50 µl (IC_50_-87,501.6 µl) compared to Test group 1 (Table 2).

**Table 2:** Effect of ASV or MAP or combination of ASV + MAP on hyaluronidase activity of N.N venom

	Test group 1 Percent inhibition of hyaluronidase activity (mean ± SEM)	Test group 2 Percent inhibition of hyaluronidase activity (mean ± SEM)	Test group 3 Percent inhibition of hyaluronidase activity (mean ± SEM)
N.N venom (200 µg) + ASV10µl (22 µg^@^)	33.0 ± 0.77	29.3 ± 1.2^$^	10.92 ± 1.4
N.N venom (200 µg) + ASV 25 µl (55 µg^@^)	35.97 ± 0.61	37.1 ± 0.71	15.42 ± 1.79
N.N venom (200 µg) + ASV 50 µl (110 µg^@^)	39.42 ± 0.19	38.2 ± 0.5	17.31 ± 1.62
N.N venom (200 µg) + ASV 100 µl (220 µg^@^)	39.59 ± 0.42	39.7 ± 0.26	21.37 ± 0.95
N.N venom (200 µg) + ASV 200 µl (440 µg^@^)	39.47 ± 0.11	40.19 ± 0.55^$^	22.24 ± 0.67^$^
N.N venom (200 µg) + ASV 300 µl (660 µg^@^)	40.73 ± 0.38^$^	41.46 ± 0.49^$^	26.8 ± 1.55^$^
N.N venom (200 µg) + MAP 100 µg	16.6 ± 1.38^$^	43.44 ± 0.41^$^	12.6 ± 1.97
N.N venom (200 µg) + MAP 200 µg	42.8 ± 1.47^$^	47.75 ± 0.19^$^	35.7 ± 1.62^$^
N.N venom (200 µg) + MAP 400 µg	52.23 ± 1.22^$^	59.9 ± 0.64^$^	50.28 ± 0.43^$^
N.N venom (200 µg) + ASV 25 µl (55 µg^@^) + MAP 100 µg	38.55 ± 0.99^$^	42.5 ± 0.38^$^	25.35 ± 1.39^$^
N.N venom (200 µg) + ASV 25 µl (55 µg^@^) + MAP 200 µg	49.06 ± 1.25^$^	49.7 ± 0.68^$^	36.81 ± 0.92^$^
N.N venom (200 µg) + ASV 25 µl (55 µg^@^) + MAP 400 µg	62.12 ± 0.76^$^	61.07 ± 0.51^$^	50.14 ± 0.34^$^

Test group 1 represents estimation of hyaluronidase activity in N.N venom after addition of ASV or MAP or combination of ASV + MAP to the substrate. There was no time gap between the additions of successive reagents; Test group 2 represents estimation of hyaluronidase activity in N.N venom where ASV or MAP or combination of ASV + MAP were incubated with N.N venom for 30 min at 37 °C prior to the addition of the substrate; Test group 3 represents estimation of hyaluronidase activity in N.N venom where N.N venom was incubated with its substrate for 90 min at 37 °C, following which ASV or MAP or the combination of ASV + MAP was added to the assay mixture; hyaluronidase activity of 200 µg N.N venom was considered as 100% and designated as venom control. All the test group values of hyaluronidase activity were compared with venom control. Test group values shown above are mean ± SEM of triplicate samples where ^$^*p* < 0.05 compared to N.N venom (200 µg) + ASV (25 µl); ^@^represents concentration of proteins in ASV based on Lowry’s method

*N.N* *Naja naja*, *MAP* methanolic extract of *Andrographis paniculata*, *ASV* anti-snake venom

**Fig. 4:**
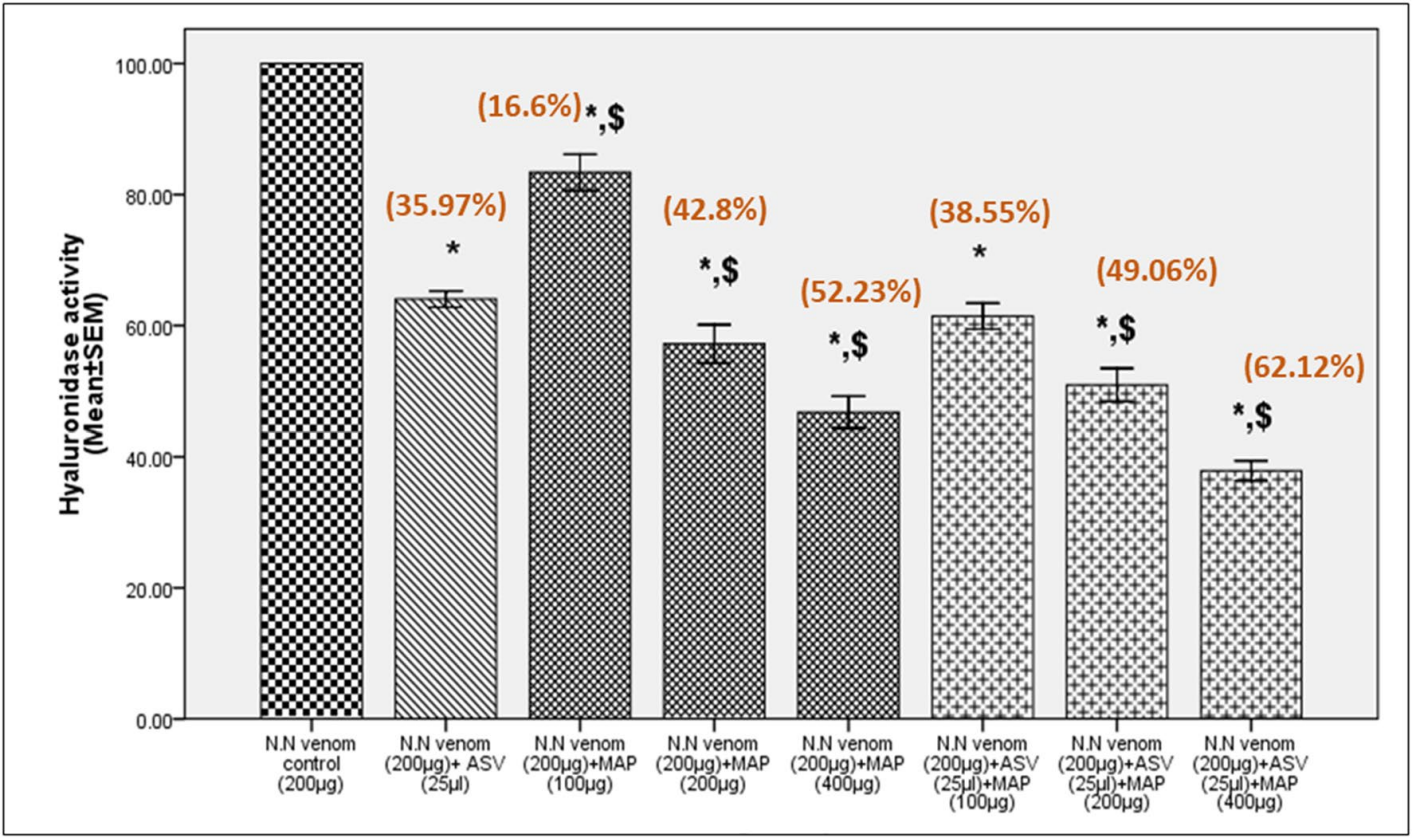
Inhibition of N.N venom hyaluronidase activity in Test group 1. Test group 1 represents estimation of hyaluronidase activity in N.N venom after addition of ASV or MAP or combination of ASV + MAP to the substrate. There was no time gap between the additions of successive reagents. All values represent mean ± SEM of three samples, where *p < 0.05 compared to N.N venom control; ^$^*p* < 0.05 compared to N.N venom (200 µg) + ASV (25 µl). Values in brackets indicate % inhibition in hyaluronidase activity compared to N.N venom control which was considered 100%; *N.N* *Naja naja*, *ASV* anti-snake venom, *MAP* methanolic extract of *Andrographis paniculata*

**Fig. 5:**
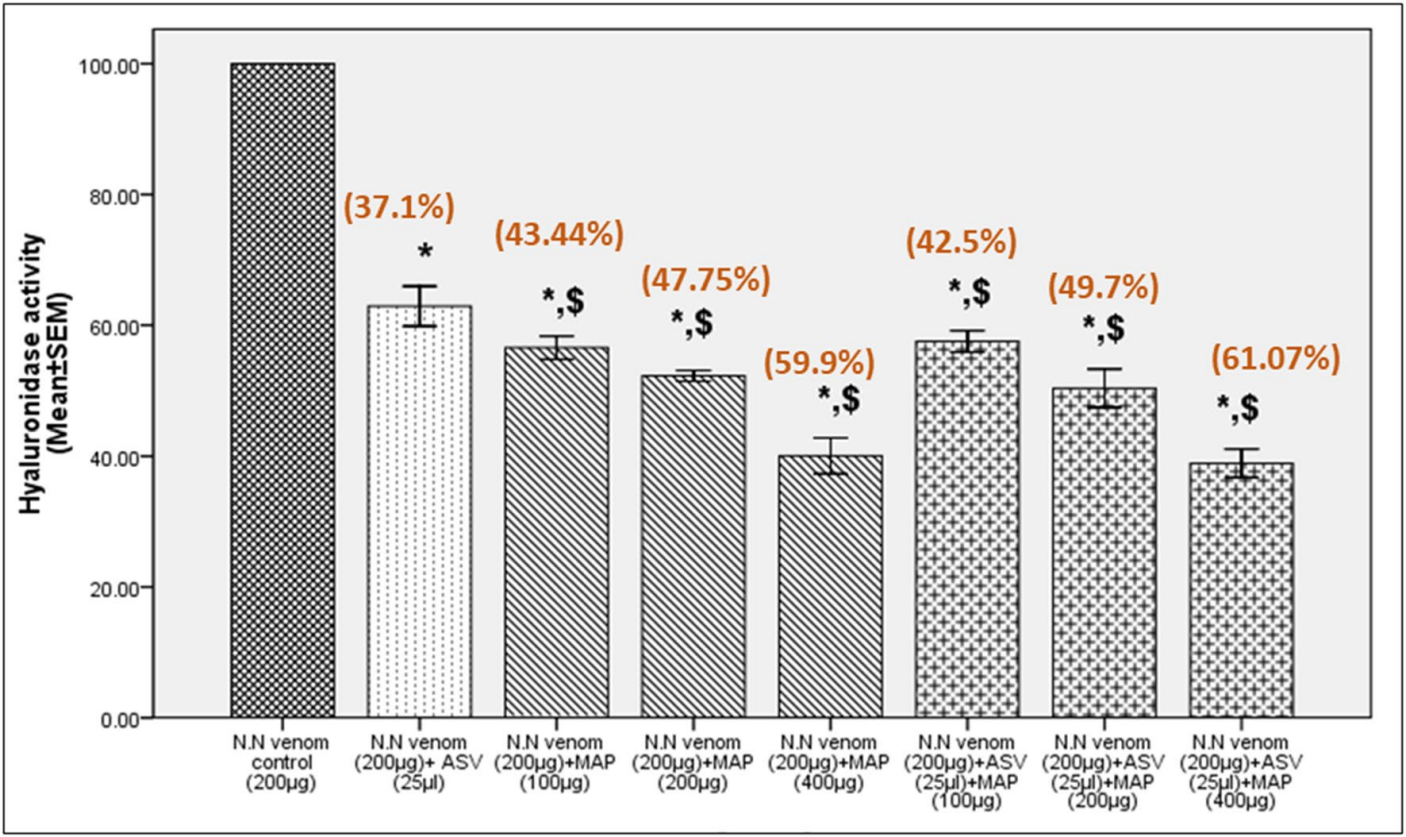
Inhibition of N.N venom hyaluronidase activity in Test group 2. Test group 2 represents estimation of hyaluronidase activity in N.N venom where ASV or MAP or combination of ASV + MAP was incubated with N.N venom for 30 min at 37 °C prior to the addition of the substrate and other reagents to the assay mixture. All values represent mean ± SEM of three samples, where *p < 0.05 compared to N.N venom control; ^$^*p* < 0.05 compared to N.N venom (200 µg) + ASV (25 µl). Values in brackets indicate % inhibition in hyaluronidase activity compared to N.N venom control which was considered 100%; *N.N* *Naja naja*, *ASV* anti-snake venom, *MAP* methanolic extract of *Andrographis paniculata*

**Fig. 6:**
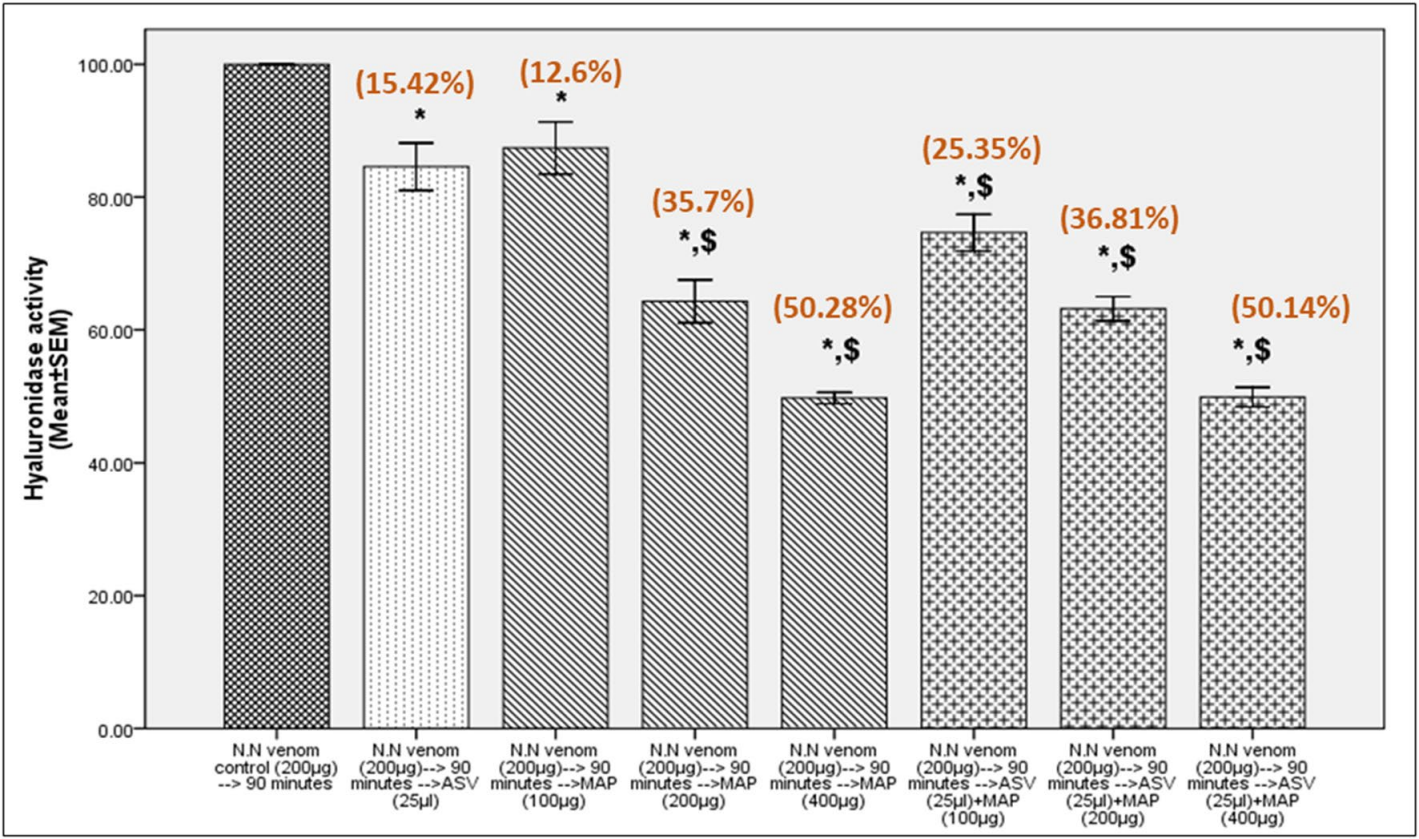
Inhibition of N.N venom hyaluronidase activity in Test group 3. Test group 3 represents estimation of hyaluronidase activity in N.N venom where venom was incubated with its substrate for 90 min at 37 °C, following which ASV or MAP or the combination of ASV + MAP was added and hyaluronidase activity was estimated. All values represent mean ± SEM of three samples, where *p < 0.05 compared to N.N venom control; ^$^*p* < 0.05 compared to N.N venom (200 µg) + ASV (25 µl). Values in brackets indicate % inhibition in hyaluronidase activity compared to N.N venom control which was considered 100%; *N.N* *Naja naja*, *ASV* anti-snake venom, *MAP* methanolic extract of *Andrographis paniculata*

In Test group I, MAP (IC_50_-326.3 µg) showed a concentration-dependent increase in the inactivation of hyaluronidase. The extent of inhibition of hyaluronidase was greater than with ASV (Table 2) when used at a ratio of 1:2, i.e., venom:MAP. Incubation of venom with MAP for 30 min prior to the addition of substrate (Test group 2, IC_50_-194.0 µg) revealed that inhibition of hyaluronidase by MAP constituents increases by 13% (Table 2) at venom:MAP ratio of 1:2 (compared to Test group 1) and the association results in the formation of stable complexes. In Test group 3, there was no significant reduction in the inhibitory activity by MAP (IC_50_-375.8 µg) constituents (Table 2) even after venom had acted on the substrate for 90 min (compared to Test group 1). This is in contrast to ASV, which lost 34% of its inhibitory capacity on incubation for 90 min. This confirms that MAP is much more effective and stable than ASV in inhibiting hyaluronidase activity in the long term. Reducing ASV concentration to 25 µl (by 92.5% than the recommended) and supplementing it with different concentrations of MAP showed a better inhibition in hyaluronidase activity in the range 39–62% in Test group 1 (Fig. 4), 43–61% in Test group 2 (Fig. 5) and 25–50% in Test group 3 (Fig. 6), compared to ASV alone.

## Discussion

India, being a large country, has a very wide geographical distribution of the N.N. species and the composition of the venom in different parts of the country varies (Shashidharamurthy et al. 2002). The fact that the commercially available antivenom showed poor inhibition of AChE activity reflects a poor match between the venom and ASV and raises questions regarding its efficacy. It is also possible that the venom used in the manufacture of ASV was poorly immunogenic with respect to AChE and hence the titer of antiacetylcholinesterase was low in ASV (Williams et al. 2019, 2011; Shabbir et al. 2014; Chotwiwatthanakun et al. 2001). This may be the reason why the ptosis which is observed in victims of Naja bite does not always respond to ASV and has to be treated with neostigmine, an AChE inhibitor (Lee et al. 2004). Increasing the concentration of ASV three times did not significantly (*p* < 0.05) change AChE inhibition. This may be explained by the Marrack’s lattice hypothesis, whereby excess antibodies do not improve antigen–antibody reaction (Matumoto 1948). In toto, the results once again underline the importance of setting up regional ASV manufacturing centers using local snake venoms for developing more effective ASVs, to address the local needs of the community. This approach has also been suggested by other studies (De Silva et al. 2016; Williams et al. 2019; Brown and Landon 2010). However, based on the present study, a more effective alternative approach of supplementing ASV with a suitable plant extract such as MAP would result in a better outcome in addressing neurotoxicity (Williams et al. 2019).

In contrast to ASV, phytochemicals from MAP completely neutralized AChE on incubation with N.N venom. The mechanism of action of MAP on AChE does not depend on antigen–antibody reactions as in the case of ASV. It may be attributed to the presence of several organic compounds such as flavonoids, terpenoids, glycosides and polyphenols in MAP (Suganthy et al. 2009; Adewusi et al. 2010; Mukherjee et al. 2007; Roseiro et al. 2012). If the human body is considered as an incubator which works at 37 °C (as used in these experiments), administration of MAP to an N.N bite victim would provide significant inhibition of AChE to mitigate neurotoxicity. When the substrate was mixed with venom and immediately followed by the addition of both ASV and MAP, the inhibition of AChE was better than when either was used alone. This demonstrates a supplementary effect between ASV and MAP in inhibiting AChE. The inhibitory effect is not additive, most probably because the phytochemicals in MAP with very large ring structures have to maneuver themselves and compete with the antibodies to bind to the enzyme.

Inhibition of hyaluronidase by ASV was only partial (41%) and it was stable for a short period of 30 min. Loss of inhibitory activity on prolonged incubation for 90 min suggests a weak interaction between the enzyme and its antibody. This may also be due to the firm binding of hyaluronidase to the substrate, making it less accessible for inhibition by the ASV. This situation represents the condition when an envenomed patient is brought to the hospital an hour-and-a-half after snake bite. The decreased inhibition of hyaluronidase by ASV (as demonstrated in Test group 3 experiments) on prolonged incubation would cause significant loss of extracellular matrix, enabling the spreading of the venom and decreasing the clinical utility of ASV.

The inhibition of hyaluronidase with MAP was quantitatively better (52% in Test group 1) than with ASV. It was also swift and stable for a prolonged period of 90 min and thus, its action was superior to that of ASV. This is probably because MAP constituents such as flavonoids can compete for the substrate-binding sites of hyaluronidase, unlike ASV which binds only to the antigenic epitopes, to form stable complexes inhibiting the enzyme (Girish and Kemparaju 2005). As in the case of AChE, the combination of ASV and MAP on hyaluronidase activity, though not additive, was better than when either was used alone. It was stable and clearly demonstrated the supplementary effect of MAP with ASV. Prolonged incubation of venom with the substrate for 90 min (Test group 3, Table), followed by the addition of ASV + MAP, did not show significant difference compared to when MAP was used alone. This could be due to the competition causing steric hindrance between the phytochemicals in MAP and the antibodies in ASV to bind to the enzyme. However, the inhibition of hyaluronidase by the combination of ASV (25 µl) + MAP (400 µg), as seen in Test group 3, was 69% higher than when ASV was used alone. This proves that MAP can work as a supplement to ASV when the concentration of the latter is drastically reduced by 92.5%.

## Conclusion

As evidenced in this study, the neutralization of AChE by ASV was poor and that of hyaluronidase unstable, leaving much to be desired. In hospitals across the world, ASVs are used with great caution and administered only when required. In case of N.N bite, ASV is generally administered only if there is evidence of neurotoxicity, manifesting as ptosis of the eyes. By the time the patient reaches the hospital and is assessed for envenomation, precious time is lost, which sometimes causes disfiguring wounds and varying degrees of muscle paralysis. While ASV has its place in treating toxicity, the fact that MAP was found to be superior to ASV in neutralizing AChE and hyaluronidase implies a new approach in dealing with N.N envenomation. Supplementing antivenom with MAP would not only reduce the antivenom given to the patient, but also reduce the chances of anaphylactic reactions, since lower doses of antivenom are known to cause fewer incidents of anaphylaxis. Using MAP alongside ASV or perhaps even prior to treatment with ASV might ensure a better outcome in snake bite, especially by reducing the local effects and systemic spreading of the venom. However, these beneficial effects need to be confirmed with in vivo experiments in animal models. Since venoms are toxic cocktails and have evolved as a multipronged strategy for survival of the snake, it is but imperative that the strategy to come out unscathed from N.N bites should also be multipronged. The efficacy of MAP in normalizing hemostatic abnormalities induced by N.N venom (Nayak et al. 2020) when viewed along with the findings of the present study gives MAP a formidable place as a potent antitoxin. This gives credence to a new approach of combining ASV with MAP to treat envenomation.
